# Grave Position at St. George's Hospital

**Published:** 1914-01-10

**Authors:** 


### GRAVE POSITION at ST. GEORGE'S HOSPITAL.

### THE CAUSES OF THE PRESENT DEADLOCK.

### Cease Personal Intrigues.

### MAKE THE HOSPITAL'S INTERESTS PARAMOUNT.

Our readers will remember that in The Hospital of the 27th ultimo, pages 337 *et seq.*, a full account was given of the proceedings at the Special Court of Governors of St. George's Hospital held on the 19th ultimo, when by a majority of thirty votes to eighteen the proposal to appoint Mr. West, one of the treasurers, as paid secretary superintendent at a total emolument, stated to be equivalent to £1,100 a year, was rejected. Under Law 29, the decision of the Special Court, rejecting the proposal to create a new "highly-paid post for the treasurer," is final and cannot be reopened. In such circumstances any further attempt to provide Mr. West with a remunerative post should have ended. It is hardly credible, but it appears to be probable that some of the medical members who are in a majority at present on the House Committee mean to make a further attempt, with Mr. West's concurrence, to reopen the question. The impropriety of any such attempt must be apparent when it is realised that Mr. West has twice resigned the treasurership and twice resumed it. We understand, with the concurrence of the majority of the House Committee, Mr. West is at present in entire charge of the administration of St. George's Hospital, and is in a position of supreme control. We venture to say that in no voluntary hospital in this country, where the management is entrusted by the Governors to a committee of impartial and independent laymen, could such an unfortunate position prevail.

#### The Mystery Explained.

The causes which have suspended the enforcement of the laws of St. George's Hospital by avoiding the appointment of a medical superintendent since July 1913 are demonstrated by the following brief summary of what has been going on since the middle of last July. At that date, as will be seen later, a Sub-committee was appointed by the House Committee to consider the proposal to create the office of a paid treasurer, and brought up a report negativing this proposal to the House Committee on July 31, 1913; the consideration of this report was postponed, in Mr. West's absence, pending his return and for other reasons, until October. On October 1 it came up on a motion for its production, when a letter was read from Mr. West to the Chairman, which amounted to the offer of his immediate resignation, and gave reasons why it was desirable it should be made operative at once. The report on the payment of the Treasurer and Mr. West's letter were postponed for consideration to the next meeting, held on October 8, 1913, when the proposal to pay the Treasurer was negatived, no one speaking or voting in favour of it.

At subsequent meetings of the House Committee, held during October, Mr. Haward, a retired member of the medical staff, moved for a Sub-committee to consider what the duties of the resident medical superintendent should be. This motion, it was understood, was made with the object of providing a way for the retention of Mr. West's services in a salaried capacity. During November, this Sub-committee prepared, and, on November 19, brought up, a report to the House Committee to retire the present Secretary, and to appoint Mr. West as Secretary at a maximum salary of £600 with allowances for house rent. The House Committee adopted the Sub-committee's recommendations, and referred them to the Finance Committee. The Finance Committee met on November 24, when they revised the proposal with reference to the present Secretary and increased the proposed emoluments to Mr. West by the addition of £150 a year for board and service. There were present at the Finance Committee Mr. Warrington Haward, Mr. Keyser, and Mr. West. Only these three members being present, one of them protested and suggested an adjournment. We are informed that Mr. West wrote the Finance Committee's report containing the proposed increase which recommended that there should not only be a Secretary but a Secretary Superintendent.

This report was submitted to a meeting of the House Committee on November 26, when Lord Arthur Hill objected to it, and its consideration was adjourned. On the following Wednesday, December 3, the matter came up again, when the adjourned report of the Finance Committee, slightly amended, was adopted. A requisition signed by the requisite number of governors was lodged to summon a Special Court on December 19. We have given this full account of the proceedings which followed the rejection of the proposal to appoint Mr. West as paid Treasurer, because they show that the schemes first to make him the paid Secretary, and subsequently to appoint him Secretary Superintendent at a high rate of remuneration, were considered from several points of view, submitted first to a special Sub-committee, next to the Finance Committee and then to the House Committee, so that the matter was fully threshed out from every point of view during a period of several weeks, before it was submitted to the decision of the governors on December 19, 1913.

#### A New Move.

The meeting of the governors on December 19 was largely attended, when the majority against the proposal to elect Mr. West the paid Secretary and Superintendent of the hospital was nearly two to one. In view of the finality of that decision under the existing laws, it was understood that the attempts to find a paid berth for one of the treasurers would cease. It was surely time that the governors and committees of the hospital, being thus freed from all these personal questions, should find themselves at liberty to devote their whole energies to the pressing business of St. George's Hospital, which included urgent and important matters, and administration. Since the retirement of Mr. Friend, the medical superintendent, in June 1913, the appointment of a gentleman to fill this office has been kept in abeyance. Some members expected that a medical superintendent would have been appointed at the meeting of the House Committee on December 31, 1913, but, instead of that, further delay was brought about by a motion by a medical member to appoint yet another Sub-committee to consider the functions of this officer. Some of the lay members pointed out the grave responsibility of longer leaving the hospital without a medical superintendent. Then, when no sufficient reasons were advanced to justify further obstruction to the regular working of the hospital, the motion was stoutly resisted in committee. The medical members, however, were in a large majority—ten of them being present and five laymen—and the motion found a majority.

### A Grave Injustice to the President.

In *The Times* of January 1, 1914, some light was thrown upon the action taken, by the following official announcement published on page 8:—

"ST. GEORGE'S HOSPITAL.

"An Independent Inquiry.

"*Princess Christian, as President of St. George's Hospital, has given directions for an independent inquiry into the charges brought by certain governors at the Court of Governors of December 19 against the administration of the hospital by the treasurers, which have led to the resignation of the two treasurers, Lord Plymouth and Mr. A. William West. In these circumstances the two treasurers have consented to withhold their resignations for the present.*"

A study of the full report of the proceedings at the Special Court of Governors on December 19 will demonstrate that no such inquiry, independent or otherwise, can be held, for the reason that no charges of any kind were "brought by certain "governors at the Court on December 19 against "the administration of the hospital by the trea- "surers." One governor at this Court used the words "medical school" instead of "medical staff," and another made certain statements which he could not have carefully verified beforehand. Such things are not of sufficient importance to justify the President to direct an independent inquiry into them. Whoever therefore is responsible for moving H.R.H. Princess Christian, as President of St. George's Hospital, in such circumstances to perpetrate an absurdity, has incurred a serious responsibility which cannot be too strongly condemned. It amounts to nothing short of a grave breach of decorum and constitutes an unjustifiable act towards the President. Here again the true inwardness of the immediate object, the drawing of a herring across the scent, is revealed, by the use of Lord Plymouth's name. Lord Plymouth is a public man who deservedly has the high respect and appreciation of all classes of the community, owing to his public work and the generosity which he has recently displayed in the interests of Londoners. Lord Plymouth's name was never mentioned at the Special Court by anyone present, nor was any reference, directly or indirectly, personally or officially, made to him or to his official duties at St. George's Hospital. We have shown that an inquiry cannot be held for the purpose given in the official notice we have quoted from *The Times*, so that notice must be either amended or withdrawn, unless, as seems to be the wiser course, it is allowed to fall into the limbo of forgotten things.

### The Cause of the Trouble.

Our readers, after perusing the facts as here given, may well feel mystified and amazed. What is the secret of these extraordinary, and in many aspects most regrettable, occurrences at St. George's Hospital? It can be stated in a few words. A month or two after Dr. Friend resigned the medical superintendentship, Mr. West, at a meeting of the House Committee in the middle of July, announced from the chair that he found he could not continue to hold the post of treasurer, as it involved too great an interference with his private business, which he could not afford to sacrifice. He asked that a small Sub-committee might be appointed to consider the future management of the hospital, and undertook, while remaining treasurer, to discharge the duties of superintendent, apart from those of resident medical officer, until suitable arrangements for carrying on the affairs of the hospital and its management were settled. This Sub-committee was appointed, as we have already described, and found itself face to face with a proposal to appoint a paid treasurer. The decision of the House Committee against this proposal we have already given. Subsequent measures to retain Mr. West's services in a paid capacity have demonstrated that these proposals have not commended themselves to those whose duty it has been to decide them.

Why have these decisions not been accepted, and why has the administration of St. George's Hospital received such scant consideration at the hands of those in charge of the Executive? The reason is, we understand, that a few of the senior members of the medical staff honestly believe that in existing circumstances it would be an advantage to retain Mr. West's services in some capacity by a payment out of hospital funds. This view is not held by eminent laymen on the House Committee nor by a majority of the governors, to whom the decision was referred at the Special Court. These gentlemen hold that the opinion of the medical members of the House Committee cannot be accepted in the best interests of St. George's Hospital, seeing that the present position of the affairs and the general administration of this hospital to-day require the services of a trained expert, possessed of the knowledge and driving force to put the whole administration of this institution upon an up-to-date basis. He must raise its annual revenue, as such a man could readily raise it, to an amount adequate to meet the necessary expenditure and to maintain it free from debt. All this is not the work of an architect like Mr. West, but of a specially trained expert, such as the other great hospitals employ.

Let Everybody put the Hospital's Interests First.

We have known Mr. West in various capacities for many years. We appreciate his good points, and his endeavour to promote the interests of the hospital. All the unfortunate circumstances of the last six months, which have mystified the governors and hospital people generally, would have been non-existent, had Mr. West decided to take the usual course and insist upon resigning the post of treasurer, when he found it necessary to announce from the chair in July that he could not continue to hold this post any longer, for a reason which secured for him the sympathy of everyone present. Had he done this he would have shown that the interests of St. George's Hospital were paramount, and that he, as treasurer, intended to uphold them. It is still open to him to adopt the course indicated. There are grave matters of pressing importance vital to the interests of St. George's Hospital, which must be faced and determined without a moment's avoidable delay. It has been our responsible duty to-day to endeavour to clear away the confusion, and to point the path which every one interested in St. George's Hospital may properly follow, because it will lead to the wisest possible solution of the present difficulties of the situation.

